# Investigation of the Antioxidant Capacity, Insecticidal Ability and Oxidation Stability of *Chenopodium formosanum* Seed Extract

**DOI:** 10.3390/ijms19092726

**Published:** 2018-09-12

**Authors:** Kai-Jen Chuang, Zong-Jiang Chen, Chih-Lun Cheng, Gui-Bing Hong

**Affiliations:** 1 School of Public Health, College of Public Health and Nutrition, Taipei Medical University, Taipei 11031, Taiwan; kjc@tmu.edu.tw; 2 Department of Public Health, School of Medicine, College of Medicine, Taipei Medical University, Taipei 11031, Taiwan; 3 Department of Chemical Engineering and Biotechnology, National Taipei University of Technology, Taipei 10608, Taiwan; d8906002@yahoo.com.tw (Z.-J.C.); ray53184@gmail.com (C.-L.C.)

**Keywords:** antioxidant, insecticidal, heat oxidation, *Chenopodium formosanum*, optimization

## Abstract

To maximize the extraction of antioxidants from *Chenopodium formosanum* seeds, the process factors, such as the ethanol concentration (0–100%), extraction time (30–180 min) and temperature (30–70 °C), for the extraction of the bioactive contents as well as the antioxidant capacity are evaluated using response surface methodology (RSM). The experimental results fit well with quadratic models. The extract was identified by GC/MS, and it was found that some active compounds had antioxidant, repellency and insecticidal activities. Various concentrations of the extract were prepared for the evaluation of the insecticidal activity against *Tribolium castaneum*, and the toxicity test results indicated that the extract was toxic to *Tribolium castaneum*, with an LC_50_ value of 354.61 ppm. The oxidative stability of the olive oil determined according to the radical scavenging activity and *p*-anisidine test demonstrates that the extract obtained from the *Chenopodium formosanum* seeds can retard lipid oxidation.

## 1. Introduction

Excessive amounts of reactive oxygen species (ROS), which are constantly generated in living organisms by aerobic organisms and exogenous sources, may cause biomolecular oxidation and generate oxidative stress that can cause diseases and disorders [1,2]. Environmental influences such as UV radiation, toxicants, pollutants and diet are also major factors in accelerating the human aging process. There is a high correlation between the aging process and oxidative stress in modern civilization. It has been recognized that the effects of oxidative stress can be reduced by some antioxidant compounds [3,4]. Antioxidant compounds play an important role in protecting the body from some diseases associated with aging [5]. In addition, natural bioactive compounds can act as alternatives to synthetic pesticides in the protection of plants against pests as natural insecticides for new insect control products and can be used to improve the oxidative stability of edible oils [6,7,8]. Both synthetic and natural antioxidants are widely used in skin care products, nutrient foods, controlling insect pests and preventing oxidative deterioration. However, synthetic antioxidants were not well accepted by customers and are restricted in many countries due to possible undesirable effects on human and animal health [9,10]. Herein, many natural plants and fruits have been investigated as sources of antioxidants.

*Chenopodium formosanum*, also known as djulis, is a native cereal plant of Taiwan that is a major source of colorants and antioxidants [11,12]. *Chenopodium formosanum* has colorful leaves, but its bright red seeds are most likely why it is called “Hung Li” in Chinese [13]. The solvent extraction process is relatively efficient and is usually applied to extract bioactive compounds from plants and fruits. Phenolic compounds are preferably extracted with ethanol, which is an environmentally friendly solvent with a high extraction efficiency and lower toxicity and cost [14]. To obtain the maximum extraction of antioxidants from *Chenopodium formosanum* seeds, the effect of the ethanol concentration, extraction time and temperature on the extraction of the bioactive contents as well as the antioxidant capacity is evaluated by using the response surface methodology (RSM). The extract of *Chenopodium formosanum* seeds based on the optimum extraction conditions was used to evaluate the insecticidal activity against *Tribolium castaneum* by toxicity bioassays as well as the oxidative stability of the olive oil by using the radical scavenging activity and the *p*-anisidine tests.

## 2. Results and Discussion

### 2.1. Model Fitting

The total phenolic content (TPC), total flavonoid content (TFC) and antioxidant capacity (FICA) experimental results of *Chenopodium formosanum* seed extracts based on the central composite design (CCD) of RSM are tabulated in Table 1. The TPC and TFC of *Chenopodium formosanum* seed extracts ranged from 3.051 to 11.861 mg GAE g^−1^ DS and from 1.265 to 3.522 mg QE g^−1^ DS, respectively. In addition, the results of the FICA assay were in the range of 0.02 to 12.092 mg EDTA g^−1^ DS. Three commonly used models (linear, quadratic and cubic) were applied to represent the experimental data, and it was found that the quadratic model was the most suitable, with the *p* value less than 0.05, as shown in Table 2. The regression models in coded factors for TPC, TFC and FICA are obtained as follows:

(1) $$\begin{array}{l} \mathrm{TPC}=11.47-1.32X_{1}-0.084X_{2}+0.11X_{3}+0.32X_{1}X_{2}+1.75\times 10^{-3}X_{1}X_{3}+0.03X_{2}X_{3}\\ -1.96X_{1}^{2}-0.14X_{2}^{2}+0.044X_{3}^{2} \end{array}$$

(2) $$\begin{array}{l} \mathrm{TFC}=3.31-0.28X_{1}-8.11\times 10^{-3}X_{2}+0.12X_{3}+0.013X_{1}X_{2}-0.14X_{1}X_{3}-3.375\times 10^{-3}\\ X_{2}X_{3}-0.58X_{1}^{2}+0.013X_{2}^{2}+0.037X_{3}^{2} \end{array}$$

(3) $$\begin{array}{l} \mathrm{FICA}=11.23-2.9X_{1}+0.25X_{2}+0.61X_{3}-0.25X_{1}X_{2}-0.49X_{1}X_{3}+0.5X_{2}X_{3}-3.42X_{1}^{2}\\ -0.45X_{2}^{2}-0.56X_{3}^{2} \end{array}$$

The analysis of variance (ANOVA) was used to test the fitness of the quadratic models by using the least squares technique; the results are listed in Table 2. The linear and quadratic terms of the ethanol concentration were significant at the level of *p* < 0.05 for all of the response values. The linear term of the temperature and the interaction between the ethanol concentration and temperature for the TFC were also significant. The *R*-squared (*R*^2^), adjusted *R*-squared (Adj. *R*^2^), *F* test and lack of fit test were estimated and demonstrated the adequacy of the constructed models [15].

### 2.2. Analysis of the Response Surfaces

**Figure 1:**
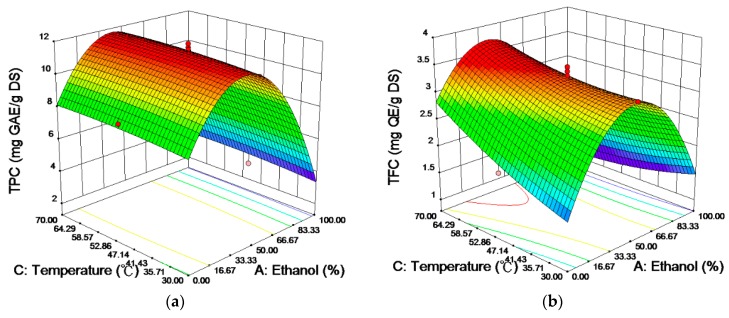

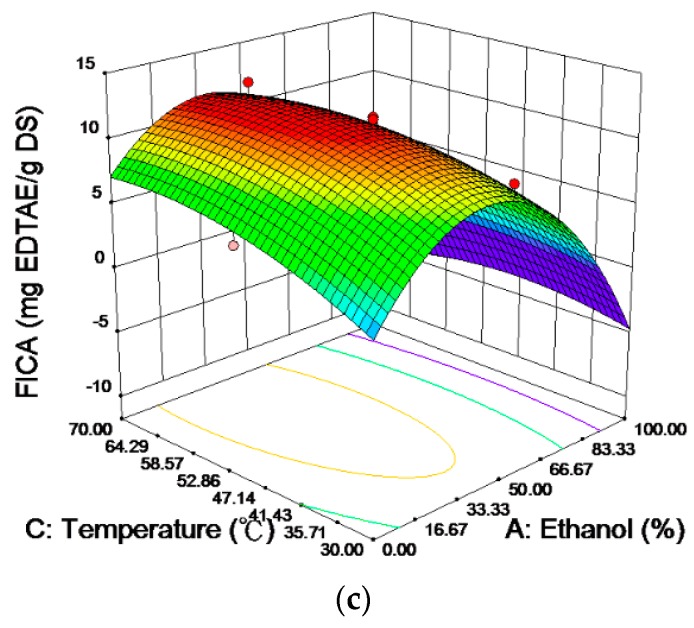
Response surface plots showing the effects of the ethanol concentration and temperature on the (**a**) TPC, (**b**) TFC, and (**c**) FICA.

**Figure 2:**
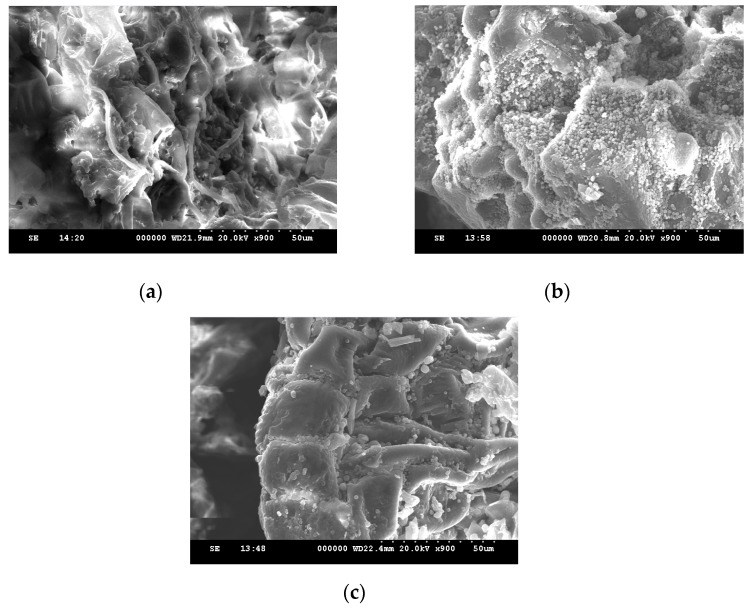
Scanning electron micrographs of *Chenopodium formosanum* samples extracted with (**a**) no solvent, (**b**) 100% water, and (**c**) 100% ethanol.

Figure 1 is the response surface plot showing the effects of the ethanol concentration/temperature on the TPC (Figure 1a), TFC (Figure 1b) and FICA (Figure 1c) of *Chenopodium formosanum* seed extracts. The ethanol concentration was the most significant process factor in regards to the response variables, and the result was consistent with the ANOVA. When the ethanol concentration increased to the medium region (33.3~48.6%), all of the response values increased to the maximum; however, they decreased when the ethanol concentration was higher than the optimal values. The polarity of the solvent plays a crucial role due to its ability to extract substances by solubilization [10]. The presence of water will lead to the polarity of the ethanol solution increasing and will also increase the contact surface area between the plant matrix and the solvent by the swelling of the plant material [16], as shown in Figure 2.

Temperature is also an important parameter, and the interaction between the ethanol concentration and temperature for the TFC was also significant (Figure 1b). This is because the plant tissues were softened, and mass transfer between the plant and solvent was accelerated to promote the diffusion of more active compounds into the solvent under higher temperature [17,18]. However, the heating process may degrade the active compounds by hydrolytic cleavage, decarboxylation or dehydrogenation [19]. Thus, the maximum temperature in the experimental design was set at 70 °C to prevent the degradation of the active components.

According to the ANOVA results from Table 2, the extraction time did not significantly contribute to the release of active components from the *Chenopodium formosanum* seeds. Moreover, the interaction between the ethanol concentration and time for the response variables was found to have no effect, except for the TPC (*p* = 0.0533, non-significantly). In general, the quantity of analytes extracted increased by increasing the extraction time, although there is a risk that the degradation of phenolic compounds may occur [20]. Therefore, the response variables of the extraction process should be influenced by the extraction time, but this impact compared to the significant effect of the ethanol concentration could be ignored.

### 2.3. Determination of the Optimal Conditions and Model Verification

The optimal conditions of the extraction process were obtained by solving the regression Equations (1)–(3) using Design Expert software based on the CCD experimental results. The predicted TPC, TFC and FICA values under the optimal conditions were 11.813 mg GAE g^−1^ DS, 3.802 mg QE g^−1^ DS and 12.580 mg EDTA g^−1^ DS, respectively. To verify the validity of the predicted values, the extraction under optimal conditions was conducted thrice for each assessment. Under the optimal conditions, the experimental values of the TPC, TFC and FICA were 12.233 ± 0.236 mg GAE g^−1^ DS, 3.628 ± 0.101 mg QE g^−1^ DS and 12.040 ± 0.495 mg EDTA g^−1^ DS, respectively. By comparing the experimental and predicted values of the response variables, low relative error values (RE < 5%) were obtained, and they verified that the fitted quadratic models are well-suited.

### 2.4. GC-MS Analysis of the Extract

Phenolic compounds include phenolic acids, flavonoids, and other active components, which can produce antioxidant, anticarcinogenic, antimutagenic, and anti-inflammatory effects as well as being harmful to insects [21]. Additionally, the optimal conditions for the TPC, TFC and FICA are quite close each other. Therefore, the extraction of *Chenopodium formosanum* seeds was conducted under the optimal TPC conditions, and the extract was obtained for the further investigation of the insecticidal activity against *Tribolium castaneum* as well as the oxidative stability of the olive oil. The extract was analyzed by using GC-MS to characterize the chemical composition. According to the analysis results in Table 3, the main component of the extract was sucrose, which accounted for 25.81%. Guaiacol (9.10%), 1,2-benzenediol (pyrocatechol) (2.18%) and hydroxy methyl furfural (5-hydroxymethylfurfural) (2.04%) have been reported to have antioxidant properties [22,23,24].

### 2.5. Toxicity Bioassays

**Figure 3:**
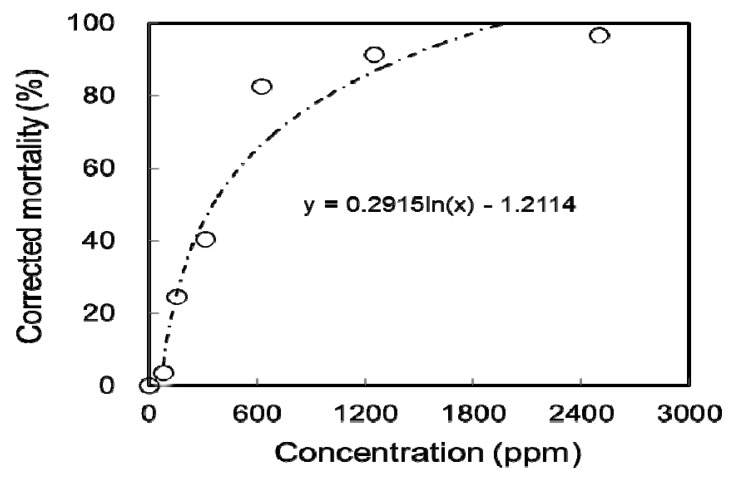
Toxicity of the extract against *Tribolium castaneum*.

Figure 3 displays the toxicity test results of *Chenopodium formosanum* seed extract against *Tribolium castaneum*. The results indicated that the extract of *Chenopodium formosanum* seeds is toxic to *Tribolium castaneum* with an LC_50_ value of 354.61 ppm. The effective insecticide of the extract was due to the active compounds such as 1,2-benzenediol, hydroxyl methyl furfural (5-hydroxymethylfurfural) and 2-heptanone, which were investigated to have repellency or insecticidal activity. Some studies have shown that 1,2-benzenediol (pyrocatechol) and 2-heptanone have repellency activity [25,26]. Tunón et al. [25] evaluated that the phenolic compound 1,2-benzenediol has repelling activity and was harmful to insects due to the two hydroxyl groups in the ortho-position. The insecticidal effect of 5-hydroxymethylfurfural against *D. melanogaster* adults is obvious, with an LD_50_ value (the lethal dose for 50% mortality) of 34.0 mg/adult [27]. The component methyl oleate accounted for 8.87% in *Chenopodium formosanum* seeds, although it is known to have oviposition-deterrent activity [13,28]. The effect of methyl oleate did not contribute directly to the toxicity test results of *Chenopodium formosanum* seed extract against *Tribolium castaneum*, and it resulted in the higher LC_50_ value (354.61 ppm) than the *Tagetes lemmonii* leaf extract (26.28 ppm) [8].

### 2.6. Oxidative Stability of Olive Oil

**Figure 4:**
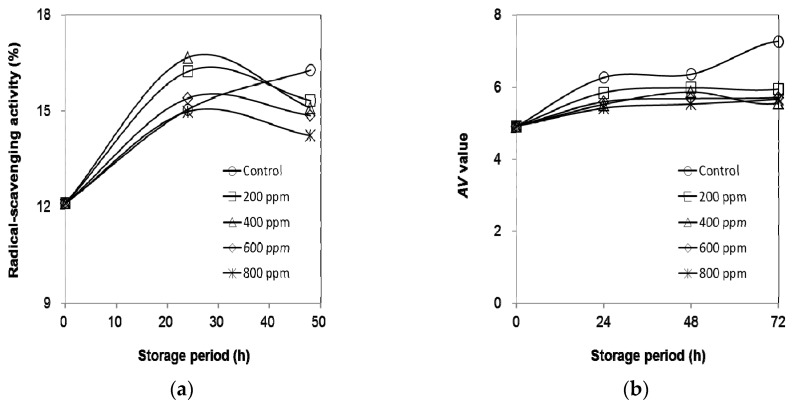
(**a**) DPPH radical scavenging activity and (**b**) AV values of olive oil supplemented with different amounts of the extract.

The DPPH radical scavenging activity and the *p*-anisidine test was used to evaluate the ability of the antioxidants in the olive oil to scavenge free radicals as well as the secondary lipid oxidation products produced during the decomposition of hydroperoxides, respectively. These experimental results are shown in Figure 4. Figure 4a shows that the radical scavenging activity results of olive oil without extracts (control sample) increase under accelerated storage. After 48 h, the radical scavenging activity decreased with the increase in the concentration of the additive, which indicates that some active components affect the stability of DPPH. The level of formation of secondary oxidation products in olive oil and the AV values of olive oil with various concentrations of extract are shown in Figure 4b. Initially, the AV values of all of the samples increased with the increase in the storage period. Obviously, the AV values of the olive oil with the extract were lower than for the control sample. A significant difference in the AV values was observed between the control and samples with the extract at 72 h. The presence of active components (guaiacol, 1,2-benzenediol and hydroxy methyl furural) showed that the antioxidant property of the extract can decrease the absorbance of DPPH. In addition, these active compounds can prevent the autoxidation of olive oil and retard lipid oxidation through the decay of lipid hydroperoxides [29,30].

## 3. Materials and Methods

### 3.1. Materials

Acetic acid (>99%), methanol (>99%), ethanol (99.8%), sodium carbonate (Na_2_CO_3_) (99.5%) and Folin-Ciocalteu’s reagent (2.0 N) were purchased from Fisher Chemical (Fair Lawn, NJ, USA). Acetic acid potassium (99%), aluminum chloride hexahydrate (AlCl_3_·6H_2_O) (99%), EDTA (99%), ferrozine, gallic acid (98%), iron (II) chloride tetrahydrate (FeCl_2_·4H_2_O) and iron (III) chloride hexahydrate (FeCl_3_·6H_2_O) were supplied by Acros (Geel, Belgium). DPPH (2,2-diphenyl-1-picrylhydrazyl) (95%) and *p*-anisidine (99%) were purchased from Alfa Aesar (Haverhill, MA, USA) and Sigma-Aldrich (St. Louis, MO, USA), respectively.

### 3.2. Extraction of Chenopodium formosanum Seeds

*Chenopodium formosanum* seeds obtained locally in Tainan, Taiwan, were ground to a fine powder. Approximately 5 g of *Chenopodium formosanum* seeds was extracted with ethanol solution, and the influential parameters such as the ethanol concentration, extraction time and temperature on the phenolic content and antioxidant capacity of the extract were determined. The extract was filtered through Whatman No. 1 filter paper, separated by centrifugation (6000 rpm), evaporated in a rotary evaporator and stored at 4 °C for further utilization. The GC/MS equipped with a Trace^™^ 1300 gas chromatograph (Thermo, Waltham, MA, USA) was used to analyze the bioactive compounds of the extract. The operating parameters were described elsewhere [8].

### 3.3. Phenolic Content Analysis

The determination of TPC of the extracts followed the method of Singleton and Rossi [31] and was determined with Folin-Ciocalteu’s reagent. Fifty microliters of the diluted extract was mixed with 200 μL of Folin-Ciocalteu’s reagent and 2 mL of distilled water. After being agitated for 5 min to mix well, 15% sodium carbonate (1 mL) was added, vortexed and incubated at room temperature for 2 h in the dark. The absorbance (760 nm) was measured by a spectrophotometer, and the TPC content was determined by the standard calibration equation. The content of total phenolics was expressed as milligrams of gallic acid equivalents per gram of dried seeds (mg GAE g^−1^ DS).

The TFC of the extracts was assessed according to the method of Chang et al. [32]. The diluted sample (0.5 mL) was separately mixed with 1.5 mL of 95% ethanol, 0.1 mL of 10% aluminum chloride, 0.1 mL of 1 M potassium acetate and 2.8 mL of distilled water. The absorbance was recorded at 415 nm by a spectrophotometer after 30 min. The content of the total flavonoids was expressed as milligrams of quercetin equivalents per gram of dried seeds (mg QE g^−1^ DS).

### 3.4. Antioxidant Capacity Analysis

The antioxidant capacity of the extract was evaluated by the ferrous ion chelating activity (FICA) based on the method of Dinis et al. [33] with minor changes. Briefly, 0.5 mL of the diluted extract was mixed with 1.9 mL of 99% methanol and 0.05 mL solution of FeCl_2_ (2 mM). After 30 s, 0.1 mL of ferrozine (5 mM) was added, and then, the mixture was vigorously shaken and left at room temperature. The absorbance of the mixture at 562 nm was measured after 10 min. The chelating activity of the extract for Fe^2+^ was expressed as milligrams of EDTA equivalents per gram of dried seeds (mg EDTA g^−1^ DS).

### 3.5. Insect Cultures and Toxicity Bioassays

*Chenopodium formosanum* seed extract was investigated for its insecticidal activity against *Tribolium castaneum*. The insects were reared using the method described by Bougherra et al. [34], and the conditions are presented in Ma et al. [8]. Adult *Tribolium castaneum* was used in the toxicity bioassays, which were based on the procedure described by Peixoto et al. [35]. Briefly, a 500 μL volume of sample extract was dropped onto a piece of filter paper, which was placed on the bottom of the Petri dish (8 cm diameter) with 20 adult insects for the toxicity test. To prevent the fast escape of the diluted extract, Parafilm was used to seal the Petri dish. In addition, some pinholes were created in the Parafilm to avoid the death of the insects from suffocation. The positive and negative controls were 0.5% of the insecticide permethrin diluted with ethanol and ethanol without permethrin, respectively. Each toxicity test was conducted in three replicates at 25 ± 1 °C and 60% relative humidity in the dark. The dead insects were recorded every 30 min, and the observed mortality (%) was evaluated after 3 h. Abbott’s formula [36] was used to correct the control mortality (%) to obtain the corrected mortality (%):

(4) $$Corrected\;mortality\;(\%)=\frac{\left(observed\;\mathrm{mortality}\;(\%)-\mathrm{control}\;\mathrm{mortality}\;(\%)\right)}{100-\mathrm{control}\;\mathrm{mortality}\;(\%)}\times 100$$

Various concentrations of the extract were prepared for the evaluation of the insecticidal activity against *Tribolium castaneum*, and the plot of the test sample concentrations versus the corrected mortality can be obtained to determine the lethal concentration of the sample to kill 50% of the insects (LC_50_ value).

### 3.6. Oxidative Stability of Olive Oil

Different concentrations (200–800 ppm) of *Chenopodium formosanum* seed extract (150 µL) were added to 50 mL of olive oil, and the samples were stored in a 100 °C oven for 3 h (pre-heated) and then cooled in a 4 °C refrigerator for 21 h. Both the heating and cooling processes were cycled for 48 h to accelerate the oxidation of the olive oil, which was based on the study of Yim et al. [37]. To investigate the oxidative rancidity of the oil, the DPPH scavenging activity was determined according to the method described by Martinez and Maestri [38], and the *p*-anisidine value (AV) was determined following the method described by Chong et al. [29] with some modifications. The detailed test procedures were explained in other literature [8].

### 3.7. Experimental Design and Statistical Analysis

The CCD with five levels and three process factors was applied to study the effects of the process factors on multiple responses, including the TPC, TFC and antioxidant capacity. Table 1 lists the original and coded values of the process factors, CCD design matrix and the corresponding response data. According to the experimental data, the fitting model was constructed using the Design Expert program (https://www.statease.com/) (version 10.0). Based on Fisher’s F-test, the analysis of variance (ANOVA) for multiple responses was used to check the adequacy of the proposed model.

## 4. Conclusions

The response surface methodology was used to evaluate the effect of the ethanol concentration, extraction time and temperature on the extraction of bioactive contents from *Chenopodium formosanum* seeds. The ethanol concentration was the most significant process factor in the extraction process. Based on the CCD experimental results, the optimal conditions of the extraction process were obtained. The relative error values between the experimental and predicted values of the response variables are less than 5%, indicating that the fitted quadratic models are adequate to predict the TPC, TFC and antioxidant capacity of the extract from *Chenopodium formosanum* seeds. The contents of the extract were further analyzed by GC/MS, and it was found that the extract containing some active compounds had antioxidant, repellency and insecticidal activities. The toxicity test results indicated that the extract of *Chenopodium formosanum* seeds is toxic to *Tribolium castaneum* with an LC_50_ value of 354.61 ppm. Additionally, the oxidative stability of olive oil during accelerated oxidation storage was enhanced due to the presence of active components in the extract. Therefore, the present study may reduce the utilization of synthetic antioxidants and pesticides as well as promote the application of natural plants and fruits.

## Figures and Tables

**Table 1:** Original and coded values of the process factors and CCD design matrix.

Independent Variables			Coded	Factor Levels				
				−1.68	−1	0	1	1.68
Ethanol concentration (%)			X_1_	0	20.3	50	79.7	100
Extraction time (min)			X_2_	30	60.4	105	149.6	180
Extraction Temperature (°C)			X_3_	30	38.1	50	61.9	70
Run	Independent varialbes			Experimental values ^1^				
	X_1_:E	X_2_:t	X_3_:T	TPC	TFC	FICA		
1	20.3	60.4	38.1	10.745	2.851	9.151		
2	20.3	60.4	61.9	11.316	3.429	9.786		
3	20.3	149.6	38.1	10.148	2.846	8.207		
4	20.3	149.6	61.9	10.238	3.405	12.092		
5	79.7	60.4	38.1	8.140	2.456	2.360		
6	79.7	60.4	61.9	8.118	2.464	2.293		
7	79.7	149.6	38.1	8.234	2.499	1.692		
8	79.7	149.6	61.9	8.931	2.499	2.336		
9	0	105	50	8.389	1.988	5.413		
10	100	105	50	3.051	1.265	0.02		
11	50	30	50	10.978	3.356	10.312		
12	50	180	50	10.752	3.261	11.901		
13	50	105	30	11.078	3.231	9.844		
14	50	105	70	11.186	3.522	11.754		
15	50	105	50	11.861	3.211	11.754		
16	50	105	50	11.124	3.400	11.571		
17	50	105	50	11.515	3.319	11.125		
18	50	105	50	11.465	3.479	11.002		
19	50	105	50	11.300	3.301	11.106		
20	50	105	50	11.643	3.161	10.408		

^1^ TPC were expressed in mg GAE g^−1^ DS. TFC were expressed in mg QE g^−1^ DS. FICA were expressed in mg EDTA g^−1^ DS.

**Table 2:** ANOVA table for the effects of the ethanol concentration, extraction time and temperature on the TPC, TFC and antioxidant capacity of the *Chenopodium formosanum* seed extracts.

Source	DF (Degree of Freedom)	SS (Sum of Squares)	F Value	*p* Value
TPC				
Model	9	80.42	51.45	<0.0001
X_1_	1	23.73	136.61	<0.0001
X_2_	1	0.097	0.56	0.4732
X_3_	1	0.17	0.97	0.3477
X_1_X_2_	1	0.83	4.80	0.0533
X_1_X_3_	1	2.45 × 10^−5^	1.41 × 10^−4^	0.9908
X_2_X_3_	1	7.08 × 10^−3^	0.041	0.8440
X_1_^2^	1	55.23	318	<0.0001
X_2_^2^	1	0.28	1.60	0.2351
X_3_^2^	1	0.028	0.16	0.6951
Linear	3	23.99	2.20	0.1278
Quadratic	3	55.59	106.68	<0.0001
Cubic	4	0.94	1.77	0.2525
Lack of fit	5	1.40	4.23	0.0698
*R*^2^ = 0.9789				
Adj *R*^2^ = 0.9598				
TFC				
Model	9	6.49	51.65	<0.0001
X_1_	1	1.07	76.87	<0.0001
X_2_	1	8.99 × 10^−4^	0.064	0.8049
X_3_	1	0.2	14.01	0.0038
X_1_X_2_	1	1.43 × 10^−3^	0.1	0.7555
X_1_X_3_	1	0.16	11.41	0.0070
X_2_X_3_	1	9.11 × 10^−5^	6.53 × 10^−3^	0.9372
X_1_^2^	1	4.87	348.84	<0.0001
X_2_^2^	1	2.54 × 10^−3^	0.18	0.6790
X_3_^2^	1	0.02	1.44	0.2583
Linear	3	1.27	1.26	0.3203
Quadratic	3	5.06	120.78	<0.0001
Cubic	4	0.056	1.00	0.4750
Lack of fit	5	0.071	1.03	0.4876
*R*^2^ = 0.9789				
Adj *R*^2^ = 0.9600				
FICA				
Model	9	294.24	10.23	0.0006
X_1_	1	114.97	35.97	0.0001
X_2_	1	0.85	0.27	0.6170
X_3_	1	5.06	1.58	0.2371
X_1_X_2_	1	0.49	0.15	0.7026
X_1_X_3_	1	1.94	0.61	0.4536
X_2_X_3_	1	1.96	0.61	0.4516
X_1_^2^	1	168.15	52.61	<0.0001
X_2_^2^	1	2.91	0.91	0.3624
X_3_^2^	1	4.49	1.40	0.2633
Linear	3	120.88	3.14	0.0545
Quadratic	3	168.96	17.62	0.0003
Cubic	4	17.57	1.83	0.2417
Lack of fit	5	30.84	27.63	0.0012
*R*^2^ = 0.9020				
Adj *R*^2^ = 0.8138				

**Table 3:** The major compounds of the extract of *Chenopodium formosanum* seeds.

Retention Time	Composition	Peak Area (%)
4.9	2-Hydroxy-2-cyclopenten-1-one	3.46
7.97	2-Heptanone	4.57
9.7	Guaiacol	9.10
14.09	1,2-Benzenediol	2.18
14.52	4-Methylbenzaldehyde	3.67
15.03	Hydroxy methyl furural	2.04
18.45	2-Methoxy-4-Vinyl phenol	3.17
26.25	Sucrose	25.81
31.33	Ethyl d-glucoside	5.50
48.94	Methyl oleate	8.87
49.16	Octadecadienoic acid	4.84
	Other	26.79
Total		100

## Abbreviations

RSM	Response surface methodology
ROS	Reactive oxygen species
TPC	Total phenolic content
TFC	Total flavonoid content
FICA	Ferrous ion chelating activity
EDTA	Ethylenediaminetetraacetic acid
